# Impact of preoperative antithrombotic discontinuation duration on clinical outcomes following hard channel surgery for chronic subdural hematoma

**DOI:** 10.3389/fneur.2025.1651267

**Published:** 2025-09-26

**Authors:** Wen-Yu Cao, Jin-ping Li

**Affiliations:** Department of Neurosurgery, Beijing Chao-Yang Hospital, Capital Medical University, Beijing, China

**Keywords:** chronic subdural hematoma, antithrombotic therapy, preoperative discontinuation, postoperative complications, neurological outcomes, health care costs, risk stratification, hard-channel puncture drainage

## Abstract

**Background:**

Chronic subdural hematoma (CSDH) is a prevalent neurosurgical disorder with an increasing global incidence, particularly among the elderly. In recent years, minimally invasive burr-hole drainage, employing a small-diameter puncture needle in conjunction with a closed drainage system, has emerged as a preferred first-line intervention. This technique is associated with several clinical advantages, including lower recurrence rates, fewer postoperative complications, reduced mortality, and shorter operative durations and hospital stays. Despite these benefits, recurrence rates still range from 5 to 30%. One unresolved issue is the impact of preoperative antithrombotic therapy on recurrence and perioperative complications, which remains controversial and poorly defined in the current literature.

**Objective:**

This study aimed to optimize preoperative antithrombotic management in patients with CSDH through a retrospective analysis.

**Method:**

A retrospective review was conducted on patients diagnosed with CSDH who underwent hard-channel puncture and drainage at Beijing Chaoyang Hospital, Capital Medical University, between December 2016 and March 2023. Patients were stratified into three groups according to their preoperative antithrombotic drug use and discontinuation status: (1) no antithrombotic use; (2) adequate discontinuation (≥7 days prior to surgery); and (3) inadequate discontinuation (<7 days). The relationship between the duration of antithrombotic discontinuation and postoperative outcomes was analyzed to inform evidence-based perioperative management strategies.

**Result:**

The results showed no significant association between preoperative antithrombotic discontinuation and major clinical outcomes. However, insufficient discontinuation was associated with a significant prolongation of hospital stay.

**Conclusion:**

Insufficient preoperative antithrombotic discontinuation did not significantly affect major clinical outcomes in patients with CSDH undergoing hard-channel puncture drainage. However, it was associated with prolonged hospital stay. These findings support individualized preoperative antithrombotic management to optimize safety and efficacy while minimizing hospitalization duration.

## Introduction

CSDH is a common neurosurgical condition that predominantly affects the elderly population, with its incidence increasing in recent years. This rise has been attributed to advancements in computed tomography (CT) imaging, population aging, widespread use of antithrombotic medications, and heightened activity levels among older adults (1). Surgical intervention remains the mainstay of treatment for CSDH.

The hard-channel puncture and drainage technique, which involves the use of a hollow screw or trocar to establish a fixed drainage channel through a burr hole, combined with a closed drainage system, has improved procedural safety and efficiency. This minimally invasive approach allows for controlled hematoma evacuation and is increasingly favored in clinical practice.

Despite these developments, the recurrence rate of CSDH remains substantial, ranging from 5 to 30%, with reoperation rates reported between 10 and 20% (2, 3). Established risk factors for recurrence include male sex, history of head trauma, antithrombotic medication use, and alcohol consumption (2). Although antithrombotic therapy is often regarded as a potential contributor to recurrence, its exact impact on morbidity remains inconclusive, and perioperative management approaches vary considerably and lack standardization (4–6).

### Objective

This retrospective study analyzed 191 patients with CSDH who underwent treatment with hard-channel drainage. The primary objective was to assess the impact of the duration of preoperative antithrombotic drug discontinuation on postoperative outcomes, including rebleeding, neurological recovery, mortality, and complication rates. Additionally, the study aimed to explore the association between discontinuation duration and clinical prognosis, thereby contributing to the optimization of perioperative management strategies for patients receiving antithrombotic therapy.

## Patients and method

### Patient selection

This retrospective study included 191 patients with CSDH who were treated at Beijing Chaoyang Hospital, Capital Medical University, between December 2016 and March 2023. The study was approved by the institutional ethics committee and conducted in accordance with the Declaration of Helsinki and its subsequent amendments.

Inclusion criteria were: (1) a diagnosis of CSDH confirmed by neuroimaging; and (2) treatment using hard-channel puncture and drainage. Exclusion criteria included: (1) presence of concomitant intracranial hemorrhage prior to surgery; and (2) incomplete or missing clinical data.

### Method

**Figure 1:**
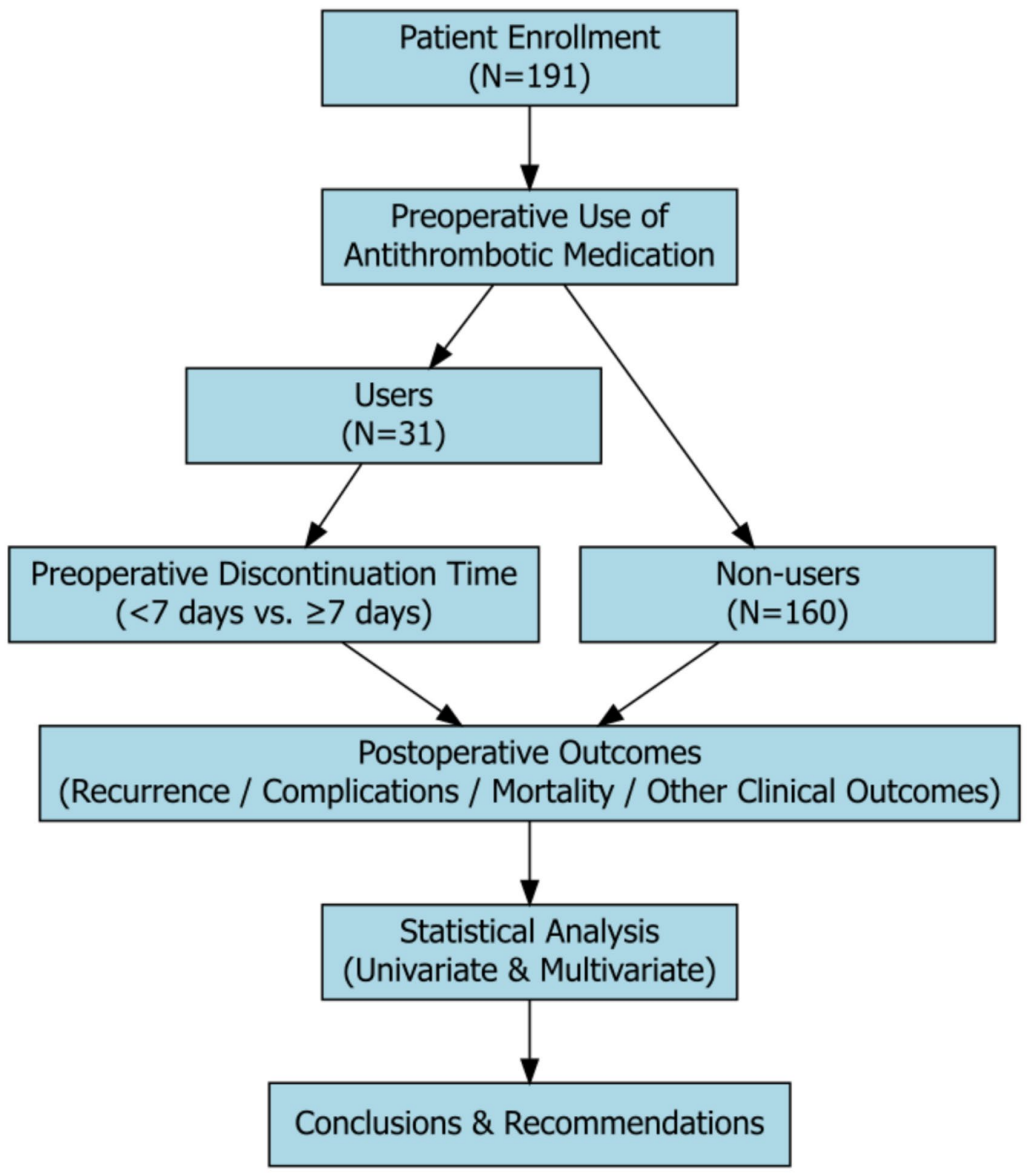
Flow chart of this research.

A flow chart illustrating the patient selection process, grouping based on preoperative antithrombotic drug discontinuation, and subsequent analysis of postoperative outcomes is presented in Figure 1. This flow chart provides a clear overview of the study design, and the distribution of patients in different groups.

#### Grouping and data collection

In this study, sufficient preoperative discontinuation of antithrombotic drugs was defined as cessation at least 7 days before surgery to evaluate perioperative safety. This threshold aligns with the 2024 American College of Cardiology/American Heart Association (ACC/AHA) perioperative cardiovascular management guidelines, which recommend stopping antiplatelet agents and vitamin K antagonists 5–7 days prior to surgery to allow for platelet function recovery and normalization of the international normalized ratio (INR) (7). Adherence to this protocol is supported by clinical evidence and established guidelines to reduce the risk of postoperative complications.

Patients were classified based on their history and preoperative status of antithrombotic medication use. Initially, participants were divided into two groups: those with a history of antithrombotic therapy (antithrombotic group) and those without (non-antithrombotic group). Within the antithrombotic group, further stratification was performed according to preoperative discontinuation status, categorized as adequate discontinuation (≥7 days) or inadequate discontinuation (<7 days). Relevant clinical data, including baseline demographics and perioperative variables, were retrospectively collected and analyzed.

The analyzed variables included patient demographics (sex and age), medical history including comorbidities and history of trauma, use and discontinuation of antithrombotic agents, preoperative statin use, and surgical approach (unilateral or bilateral). Pre- and postoperative Glasgow Coma Scale (GCS) and modified Rankin Scale (mRS) scores, and preoperative Markwalder Grading Scale (MGS) scores were collected. Imaging included pre- and postoperative hematoma thickness and the presence of hematoma septation, defined as internal partitions within the hematoma cavity indicating multiple layers of blood accumulation. Postoperative use of urokinase was recorded. Postoperative complications, such as rebleeding, infection, seizures, embolism, and thrombosis, were documented. Recurrence within 3 months, in-hospital mortality, length of hospital stay, and hospitalization costs were also included in the analysis.

#### Surgical approach and perioperative management

All patients underwent hard-channel puncture and drainage for the treatment of CSDH. The procedure was performed under local anesthesia, with the puncture site preoperatively determined by CT or MRI imaging at the point of maximal hematoma thickness. Following local disinfection and infiltration anesthesia, a small skin incision was made. A puncture needle connected to an electric drill was then used to access the hematoma cavity, with care taken to avoid injury to critical neurovascular structures.

Once the skull and dura mater were successfully penetrated, an external drainage catheter was inserted to allow immediate evacuation of the hematoma. The subdural cavity was irrigated with saline until the outflow was clear, ensuring adequate hematoma removal. A follow-up head CT was routinely performed on postoperative day 1 to assess residual hematoma volume. In cases where significant clot remained, urokinase was administered locally into the hematoma cavity to promote fibrinolysis and facilitate drainage. The drainage catheter was removed based on neurological recovery and radiological findings.

#### Study endpoints and follow-up

1. Primary outcomes: rebleeding requiring reoperation and in-hospital mortality.
2. Secondary outcomes: postoperative complications, including intracranial infection, rebleeding, seizures, and deep vein thrombosis; neurological recovery assessed by the mRS; length of hospital stay; and hospitalization costs.

### Statistical analysis

All statistical analyses were conducted using R software. Associations between recurrence, in-hospital mortality, postoperative complications, and history of antithrombotic medication use were evaluated. Categorical variables were compared using Pearson’s chi-square test or Fisher’s exact test when subgroup sizes were small (*n* < 40). Continuous variables were assessed for normality, with group comparisons performed using t-tests or Mann–Whitney U tests for small samples (*n* < 40), and Kruskal–Wallis tests for comparisons among multiple groups. Pairwise comparisons were performed following a significant Kruskal–Wallis test to identify which groups differed. Wilcoxon rank sum tests with continuity correction were used for the pairwise comparisons. *P* values were adjusted for multiple testing using the Bonferroni method to control for Type I error.

For categorical outcomes, univariate logistic regression was used to identify potential risk factors, with significant variables subsequently included in multivariate logistic regression models. For continuous outcomes, univariate linear regression analyses were performed, and variables with significant associations were entered into multivariate linear regression models.

## Results

A total of 191 patients were included in this study, with a median age of 75 years (interquartile range [IQR]: 65–82 years); 77.0% (147/191) were male. At diagnosis, 31 patients (16.2%) had a history of antithrombotic therapy: 25 received single-agent therapy, 6 received dual therapy, and 160 had no history of antithrombotic therapy. A history of head trauma was reported in 126 patients (66.0%). Comorbidities included hypertension (48.2%), diabetes mellitus (17.3%), hyperlipidemia (16.8%), cardiac disease (20.9%), cerebrovascular disease (30.4%), and malignancy (5.2%). Bilateral chronic subdural hematoma (CSDH) was observed in 56 patients (29.3%), of whom 29 (15.2%) underwent bilateral hard-channel puncture drainage. Postoperatively, 96 patients (50.3%) received urokinase therapy (Table 1).

**Table 1:** Baseline demographic, clinical, and radiological characteristics of the study population (*n* = 191).

Characteristic	Value
Age (Median [IQR])	75 [65–82]
Sex, *n* (%)	
Male	147 (77.0)
Female	44 (23.0)
Trauma, *n* (%)	126 (66.0)
Medical history, *n* (%)	
Hypertension	92 (48.2)
Diabetes	33 (17.3)
Hyperlipidemia	32 (16.8)
Heart disease	40 (20.9)
Malignant tumor	10 (5.2)
Cerebrovascular disease	58 (30.4)
Medication history, *n* (%)	
Statins	22 (11.5)
Antithrombotic medication	31 (16.2)
No antithrombotic medication	160 (83.8)
Single antithrombotic medication	25 (13.1)
Two antithrombotic medications	6 (3.1)
Unilateral and bilateral (%)	
Unilateral	135 (70.7)
Bilateral	56 (39.3)
Urokinase	96 (50.3)
Length of hospital stay (Median [IQR])	10 [8–13]
Hospitalization Expenses (USD) (Median [IQR])	1315.86 [IQR: 949.15–2018.73]
Radiological characteristics	
Preoperative hematoma thickness (total, mm) (Median [IQR])	28.0 [22.40–35.40]
Postoperative hematoma thickness (total, mm) (Median [IQR])	6.5 [0–11.55]
Midline shift (mm) (Median [IQR])	7.2 [4.65–10.05]
Hematoma separation	98 (51.3)

Values are presented as mean ± standard deviation (SD) for continuous variables and number (%) for categorical variables. Hematoma separation refers to internal septation visible on preoperative neuroimaging. Unilateral/bilateral indicates whether the CSDH was present on one or both sides.

### Radiological findings

The median hematoma absorption rate was 0.76 (IQR: 0.59–1.00). The median midline shift was 7.2 mm (IQR: 4.65–10.05 mm). The median preoperative hematoma thickness (combined bilateral measurement) was 28.0 mm (IQR: 22.40–35.40 mm), which was reduced to 6.5 mm (IQR: 0–11.55 mm) postoperatively. Hematoma separation was observed in 98 patients (51.3%) (Table 1).

#### Recurrence

The recurrence rate was 3.7% (7 cases), all of which required reoperation. Table 1 presents a comparison between patients with and without recurrence. A history of antithrombotic medication use was not significantly associated with recurrence (*p* = 0.717) (Table 2), with only one recurrent case observed among patients with such a history. This finding differs somewhat from previous studies, potentially reflecting variations in treatment protocols. Furthermore, logistic regression analysis did not identify any significant risk factors for recurrence.

**Table 2:** Clinical outcomes by duration of preoperative antithrombotic discontinuation.

Outcome	No (*n* = 160)	<7 (*n* = 21)	≥7 (*n* = 10)	*p*-value
Recurrence (%)	6 (3.8)	1 (4.8)	0 (0.0)	0.717
Death (%)	5 (3.1)	2 (9.5)	0 (0.0)	0.318
Complication (%)	11 (6.9)	3 (14.3)	0 (0.0)	0.325
mRS deterioration (%)	10 (6.3)	2 (9.5)	1 (10.0)	0.557

Values are presented as number of cases (%). Patients were categorized into three groups: no antithrombotic use (*n* = 160), <7 days of discontinuation (*n* = 21), and ≥7 days of discontinuation (*n* = 10). *P-*values were calculated using Fisher’s exact test or chi-square test, as appropriate. Statistical significance was set at *p* < 0.05.

#### Death

The overall mortality rate was 3.7% (7 cases). No significant association was observed between antithrombotic medication use or short discontinuation duration and mortality (*p* = 0.318) (Table 2). Multivariable analysis showed that heart disease (*p* = 0.031) was independently associated with increased mortality risk (Table 3).

**Table 3:** Multivariate logistic regression analysis for death.

Variable	Z	OR (95% CI)	*p* value
Heart disease	2.16	12.46 (1.65–256.53)	0.031

Z values, odds ratios (OR) with 95% confidence intervals (CI), and *p*-values are shown. Statistical significance was set at *p* < 0.05.

#### Complications

Among all patients, postoperative complications included intracranial hemorrhage in 2 cases (1.0%), seizures in 2 (1.0%), infarction in 1 (0.5%), heart failure in 6 (3.1%), pneumonia in 9 (4.7%), wound infection in 1 (0.5%), urinary tract infection in 1 (0.5%), and lower extremity venous thrombosis in 3 cases (1.6%). The duration of preoperative antithrombotic drug discontinuation was not associated with an increased risk of complications (*p* = 0.325) (Table 2). However, antibiotic use (*p* < 0.001) and MGS (*p* = 0.014) were significantly associated with an increased risk of postoperative complications (Table 4).

**Table 4:** Multivariate logistic regression analysis for complication.

Variable	Z	OR (95% CI)	*p* value
Antibiotic	3.56	1.35 (1.15–1.60)	<0.001
MGS	2.47	3.24 (1.32–8.80)	0.014

Z values, odds ratios (OR) with 95% confidence intervals (CI), and *p*-values are shown. Statistical significance was set at *p* < 0.05.

#### Deterioration of neurological function

Postoperative neurological deterioration occurred in 13 patients. The duration of preoperative antithrombotic drug discontinuation was not associated with neurological outcomes (*p* = 0.557) (Table 2). Multivariable analysis showed that heart disease (*p* = 0.007) and postoperative complications (*p* < 0.001) were independent predictors of worsening mRS scores (Table 5).

**Table 5:** Multivariate logistic regression analysis for mRS deterioration.

Variable	Z	OR (95% CI)	*p* value
Heart disease	2.69	11.63 (2.20–94.03)	0.007
Complication	3.95	226.69 (20.91–5346.62)	<0.001

Z values, odds ratios (OR) with 95% confidence intervals (CI), and *p*-values are shown. Statistical significance was set at *p* < 0.05.

### Length of hospitalization and cost

Among the 191 patients with CSDH, the median length of hospital stay was 10 days (IQR, 8–13 days), and the median hospitalization cost was $1315.86 (IQR, $949.15–$2018.73). A history of antithrombotic medication use was not significantly associated with length of stay (*p* = 0.060) (Table 6).

**Table 6:** Association of preoperative antithrombotic discontinuation time with hospital stay and expenses.

Outcome	Chi-squared	df	*p* value
Hospital stay	5.62	2	0.060
Hospitalization expenses	15.89	2	<0.001

Chi-squared values and degrees of freedom (df) are shown. Outcomes were compared among groups using the Kruskal–Wallis rank sum test. Statistical significance was set at *p* < 0.05.

**Figure 2:**
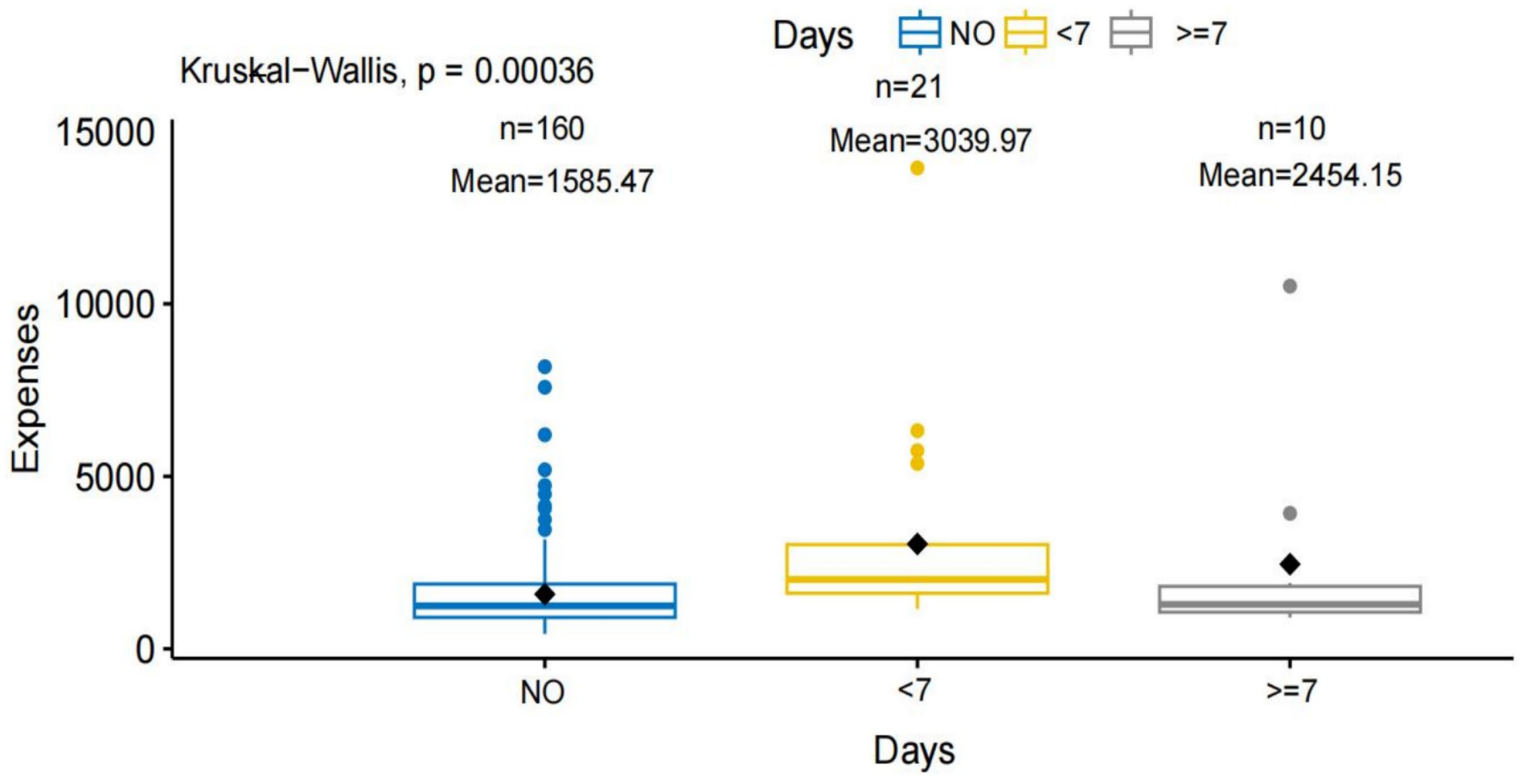
Comparison of hospitalization expenses according to preoperative antithrombotic drug discontinuation duration. This figure shows the impact of different preoperative antithrombotic drug discontinuation durations on hospitalization expenses. The x-axis (“Days”) is divided into three groups: NO, No history of antithrombotic drug use (*n* = 160, mean expense = 1585.47). <7, Antithrombotic drugs discontinued less than 7 days before surgery (*n* = 21, mean expense = 3039.97). ≥7, Antithrombotic drugs discontinued 7 or more days before surgery (*n* = 10, mean expense = 2454.15). The y-axis represents total hospitalization expenses. Black diamonds indicate the mean for each group. A Kruskal-Wallis test indicates a statistically significant difference in expenses among the groups (*p* = 0.00036).

**Figure 3:**
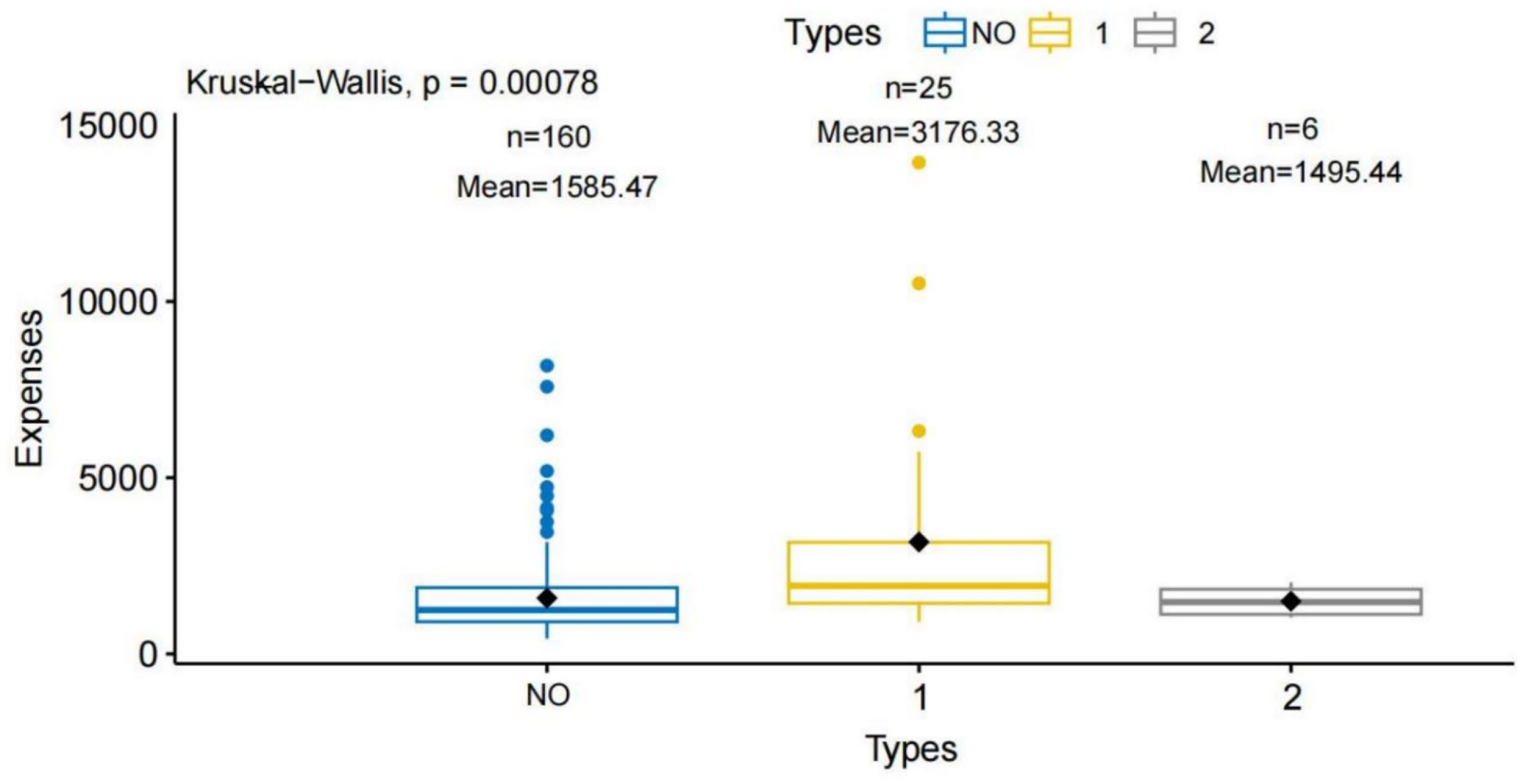
Comparison of hospitalization expenses according to the number of antithrombotic drug types used. This figure illustrates the relationship between the number of types of antithrombotic drugs used and hospitalization expenses. The x-axis represents the number of antithrombotic drug types: NO, No antithrombotic drug use (*n* = 161, mean expense = 1585.47). 1, Use of a single type of antithrombotic drug (*n* = 25, mean expense = 3176.33). 2, Use of two types of antithrombotic drugs (*n* = 6, mean expense = 1495.44). The y-axis indicates total hospitalization expenses. Black diamonds show the group means. The Kruskal-Wallis test reveals a statistically significant difference in expenses among the groups (*p* = 0.00078).

Kruskal–Wallis analysis revealed that patients with insufficient preoperative discontinuation of antithrombotic therapy (<7 days) had higher hospitalization costs than other groups (*p* < 0.001) (Figure 2; Table 6). Pairwise Wilcoxon rank sum tests with Bonferroni correction showed that these patients had significantly higher hospitalization expenses than those who did not use antithrombotic drugs (adjusted *p* < 0.001). No significant differences were observed between the other groups (adjusted *p* > 0.05) (Table 7). Hospitalization costs also varied by antithrombotic therapy type (*p* < 0.001) (Figure 3). Preoperative use of one antithrombotic drug was associated with significantly higher hospitalization expenses compared with no preoperative use (adjusted *p* < 0.001). No significant difference was observed between other groups (adjusted *p* > 0.05) (Table 8).

**Table 7:** Pairwise comparisons of hospitalization expenses (Expenses) and hospital stay (Stay) among groups with different preoperative antithrombotic discontinuation times.

Group	*n*	vs. 0 (Expenses)	vs. 1 (Expenses)	vs. 2 (Expenses)	vs. 0 (Stay)	vs. 1 (Stay)	vs. 2 (Stay)
0	160	—	<0.001	1	—	0.15	0.40
1	21	<0.001	—	0.11	0.15	—	1
2	10	1	0.11	—	0.40	1	—

*P* values were calculated using the Wilcoxon rank sum test with Bonferroni correction. Group 0, 1, and 2 represent patients with no antithrombotic use (*n* = 160), discontinuation < 7 days (*n* = 21), and discontinuation ≥ 7 days (*n* = 10), respectively. “—” indicates comparison of a group with itself. Statistical significance was set at *p* < 0.05.

**Table 8:** Pairwise comparisons of hospitalization expenses by type of preoperative antithrombotic drug.

Comparison	Adjusted *p* value
No preoperative drug vs. 1 drug	<0.001
No preoperative drug vs. 2 drugs	1
1 drug vs. 2 drugs	0.35

*P* values were calculated using pairwise Wilcoxon rank sum tests with continuity correction and adjusted for multiple comparisons using the Bonferroni method. Groups are defined as follows: no preoperative drug, no antithrombotic drug; 1 drug, 1 type of preoperative antithrombotic drug; 2 drugs, 2 types of preoperative antithrombotic drugs. Statistical significance was set at *p* < 0.05.

Multivariate linear regression identified antibiotic use (*p* < 0.001), high-level antibiotics (*p* < 0.001), duration of antithrombotic drug discontinuation (*p* = 0.047), and hematoma separation (*p* = 0.035) as independent predictors of prolonged hospitalization (Table 9). Length of hospital stay (*p* < 0.001), mortality (*p* < 0.001), and type of antithrombotic drugs (*p* = 0.004) were independently associated with higher hospitalization costs (Table 10).

**Table 9:** Multivariate linear regression analysis for length of hospital stay.

Variable	Estimate	Std. Error	t value	*p* value
antibiotic	0.68	0.11	6.18	<0.001
High level antibiotics	0.75	0.18	4.25	<0.001
Antithrombotic drugs time1	2.00	1.00	2.00	0.047
Hematoma separation	1.28	0.60	2.13	0.035

Coefficients (Estimate), standard errors (Std. Error), t-values, and *p*-values are shown. Antithrombotic drugs time1 refers to patients with insufficient preoperative discontinuation of antithrombotic drugs (compared to no use). Statistical significance was set at *p* < 0.05.

**Table 10:** Multiple linear regression analysis of hospitalization expenses.

Variable	Estimate	Std. Error	t.value	*p*.value
Hospital stay	94.83	20.80	4.56	<0.001
Death	2316.88	682.27	3.40	<0.001
Types of antithrombotic drugs1	1708.59	584.35	2.92	0.004

Coefficients (Estimate), standard errors (Std. Error), t-values, and *p*-values are shown. Types of antithrombotic drugs: 0, no preoperative antithrombotic therapy; 1, single antithrombotic agent; 2, two antithrombotic agents. Statistical significance was set at *p* < 0.05.

## Discussion

This study showed that insufficient preoperative discontinuation of antithrombotic drugs did not significantly affect major clinical outcomes. Patients who discontinued antithrombotic agents for ≥7 days before surgery had lower hospitalization costs. However, after adjusting for confounding factors, multivariate analysis showed that insufficient preoperative discontinuation was not an independent predictor of hospitalization costs. It was, in contrast, an independent predictor of prolonged hospital stay.

In recent years, hard-channel puncture and drainage has gained increasing popularity in the management of CSDH owing to its simplicity, versatility, and use of a closed drainage system. Although burr-hole drainage remains the conventional surgical approach, it has been associated with higher recurrence rates and less consistent drainage (8). Some studies have suggested that the hard-channel technique may reduce postoperative recurrence and complications (9). However, large-scale prospective trials are warranted to validate these potential advantages.

In this study of hard-channel puncture drainage for CSDH, the postoperative recurrence rate was 3.7%, which is lower than the 5–30% reported in previous literature (3). Several factors may account for this difference: the application of strict inclusion criteria, excluding patients with concurrent intracranial hemorrhage or incomplete clinical data; effective hematoma evacuation achieved through the combination of hard-channel puncture and closed drainage; timely postoperative follow-up and the adjunctive use of urokinase; and the relatively short 3-month follow-up period, which may have underestimated late recurrences. Therefore, prospective studies with extended follow-up periods and direct comparisons of surgical techniques are warranted to validate these findings.

The association between antithrombotic therapy and CSDH recurrence remains controversial. Several studies, including those by Wada et al., Froster et al., and a 2017 analysis on warfarin use, have suggested a potential link between the use of antiplatelet or anticoagulant agents and an increased risk of CSDH recurrence (2, 10). A 2018 meta-analysis and a subsequent study by Wang et al. in 2019 further supported this association (11, 12). The influence of antiplatelet drugs may depend on the timing of preoperative withdrawal. Although clinical guidelines recommend discontinuation at least 7 days before surgery to match the platelet lifespan, some evidence indicates that shorter withdrawal durations may still be safe and effective (10). Given that approximately 40% of patients with CSDH have a history of antithrombotic drug use, this remains an important consideration in perioperative decision-making (12).

Perioperative antithrombotic drug management strategies for CSDH treatment vary significantly across countries. This variation may stem from the emphasis on individualized patient risk assessment, as well as the current lack of high-quality, evidence-based guidelines for antithrombotic drug management.

### Relationship between preoperative antithrombotic drug discontinuation and postoperative hematoma recurrence

Previous studies have proposed that the primary mechanisms underlying CSDH recurrence involve persistent exudation and rebleeding processes (11). Antithrombotic agents may exacerbate these mechanisms by altering coagulation function, increasing vascular permeability, and complicating hematoma morphology, thereby potentially impairing effective drainage (10, 13). However, the present study found no significant association between preoperative antithrombotic drug use and postoperative hematoma recurrence. Further subgroup analysis revealed no statistically significant differences in recurrence rates among patients who discontinued antithrombotic therapy for <7 days, those with discontinuation ≥7 days, and those without a history of antithrombotic use.

Although previous studies have indicated higher recurrence rates in patients receiving dual antithrombotic therapy—such as a combination of anticoagulants and antiplatelet agents—or multiple antithrombotic medications compared to single-agent therapy, this association was not observed in the present study (14, 15). Furthermore, prior research has suggested that antithrombotic therapy is not an independent risk factor for recurrence in CSDH patients undergoing twist-drill drainage (1). Instead, male sex and bilateral hematomas were identified as key predictors of recurrence (1). In contrast, our study did not identify any significant predictors of recurrence among patients treated with hard-channel puncture and drainage.

### Relationship between preoperative antithrombotic drug withdrawal and postoperative mortality

The postoperative mortality rate in this study was 3.7%. Analysis of factors associated with death revealed no significant correlation between preoperative antithrombotic drug use and postoperative mortality. Our findings indicate that a history of heart disease is an independent predictor of in-hospital mortality. Analysis of fatal cases revealed that the primary causes of death were pneumonia and heart failure. These results underscore the importance of perioperative management in patients undergoing hard-channel drainage for CSDH. Preventive strategies should focus on minimizing pulmonary infection risk. In elderly patients, thorough preoperative assessment and optimization of cardiac function are essential to reduce postoperative mortality.

### Relationship between preoperative antithrombotic drug discontinuation and postoperative complications

This study found no significant association between preoperative antithrombotic drug use and the incidence of postoperative complications. Among the various complications related to chronic subdural hematoma, acute postoperative hemorrhage is one of the most severe (16). However, only two cases (1.0%) of acute postoperative hemorrhage occurred in this cohort, both of which were successfully managed with active intervention. Additionally, three patients (1.6%) developed postoperative lower extremity deep vein thrombosis, with no significant correlation to preoperative antithrombotic therapy. Previous studies have reported a higher incidence of postoperative thrombotic events in patients with a history of antithrombotic medication use, possibly due to advanced age and prolonged postoperative immobilization, with most events occurring within the first postoperative week (14). However, in our cohort, no significant association was found between insufficient preoperative discontinuation of antithrombotic medications and postoperative complications. Multivariate analysis showed that longer duration of postoperative antibiotic therapy was an independent predictor of postoperative complications. Higher preoperative MGS scores were also independently associated with complications. Perioperative management is critical. It should include infection monitoring and optimization of neurological status to reduce complications.

### Relationship between preoperative antithrombotic discontinuation and neurological outcomes

Antithrombotic therapy was not significantly associated with neurological outcomes. Heart disease was identified as an independent predictor of mRS deterioration. Postoperative complications also independently predicted worse neurological outcomes. Therefore, careful perioperative management is essential, with particular attention to patients with heart disease to improve postoperative neurological outcomes.

In addition to neurological outcomes, we evaluated factors affecting the length of hospital stay. Univariate analysis using the Kruskal–Wallis test showed no significant association between preoperative discontinuation duration and hospital stay. However, multivariate analysis identified insufficient preoperative discontinuation, postoperative antibiotic use, high-level antibiotics, and hematoma separation as independent predictors of prolonged hospitalization. Previous studies reported no significant effect of preoperative antithrombotic compliance on hospital stay (17). In contrast, our results indicate that insufficient discontinuation of antithrombotic drugs prolongs hospitalization.

These results suggest that optimizing the timing of preoperative antithrombotic discontinuation may help reduce hospital stay. Careful postoperative management of infection and hematoma-related complications is also important. Future multicenter studies with larger cohorts are needed to explore the impact of different preoperative discontinuation strategies on hospital stay and patient outcomes.

### Relationship between preoperative antithrombotic discontinuation and hospital stay and costs

Patients who did not discontinue preoperative antithrombotic medications for at least 7 days incurred higher hospitalization costs (Figure 2). However, multivariate analysis adjusting for confounding factors showed that preoperative antithrombotic discontinuation duration was not an independent predictor of costs. Hospitalization expenses were influenced by the type of antithrombotic therapy. Hospitalization costs were higher in patients receiving single-agent therapy than in those without a history of antithrombotic drug use. Additional factors affecting hospitalization costs included in-hospital death and length of hospital stay. These findings indicate that hospitalization costs are influenced by patient characteristics and clinical outcomes. Careful perioperative management may help optimize resource use and control costs.

These findings underscore the importance of individualized perioperative management of antithrombotic therapy. Insufficient preoperative discontinuation was associated with prolonged hospital stay. Careful planning of withdrawal timing is essential. Medication type, postoperative management, and patient-specific factors should also be considered. These measures may help optimize hospitalization and resource utilization while ensuring patient safety.

## Limitation

This study has several limitations. First, its retrospective, single-center design and relatively small sample size may limit the generalizability of the findings and reduce statistical power. Second, the retrospective nature introduces potential selection and information biases. Third, the relatively short follow-up period may underestimate long-term outcomes and recurrence rates. Fourth, the lack of a control group undergoing conventional burr-hole drainage precludes direct comparisons regarding surgical efficacy and safety. Additionally, intraoperative variables, specific antithrombotic regimens, and patient adherence to follow-up were not comprehensively assessed.

## Conclusion

The results indicated that the duration of preoperative antithrombotic discontinuation did not significantly affect postoperative clinical outcomes, suggesting that a uniform 7-day discontinuation period may not be strictly necessary. However, further analysis revealed that insufficient preoperative antithrombotic discontinuation significantly prolonged hospital stay. Based on these findings, individualized preoperative antithrombotic management is recommended. Such strategies should aim to optimize therapeutic efficacy and safety while minimizing length of hospitalization, with management tailored to each patient’s clinical condition.

## Data Availability

The raw data supporting the conclusions of this article will be made available by the authors, without undue reservation.
